# Supplementary material for: Understanding Human Mobility from Twitter
Source: PLoS One. 2015 Jul 8;10(7):e0131469. doi: 10.1371/journal.pone.0131469 (PMC4496063; doi:10.1371/journal.pone.0131469)
Supplement: S1 Text — (PDF) [file pone.0131469.s001.pdf]

Understanding Human Mobility from Twitter

Raja Jurdak*, Kun Zhao*, Jiajun Liu*, Maurice AbouJaoude#,
Mark Cameron*, David Newth*
*CSIRO, Australia
American University of Beirut, Lebanon

December 12, 2014

Supplementary Material

S1 Statistics of tweeting

In this section, we report statistics which capture usage patterns of Twitter. We find that the distribution of the number of tweets among users in our dataset follows a fat-tailed distribution, as shown in Figure S1(a). The result indicates that the frequency of tweeting is not homogeneous across the population; it exhibits an 80/20 effect, where a majority of registered Twitter users only contribute a small number of tweets and most tweets are posted by only a small number of frequent users. As for other technologies, there is an inherent effect that geotagged tweets can provide fine-grained data for heavy users and coarser data for lighter users. This should be considered for individual-based modelling of mobility, but on a population level, the observed dynamics still hold as in previous studies.

Next, we explore the sensitivity of our observations on mobility patterns to the number of tweets from users. Figure S1(b) shows the displacement distribution Δr separately for user groups based on the number of available tweets N in the dataset. We observe the same patterns as for the entire population in terms of movement modes. The main trend is the Twitter users with a higher N tend to have shorter steps. This is expected as their higher

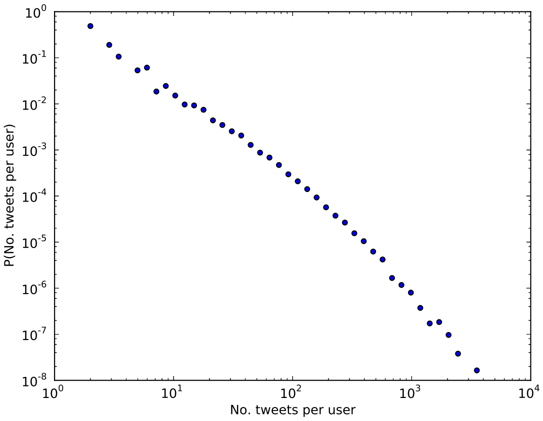

(a)

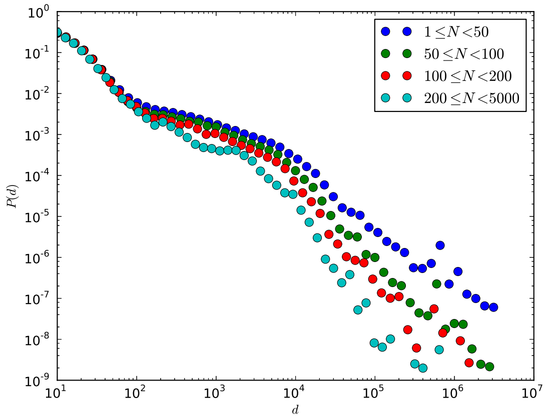

(b)

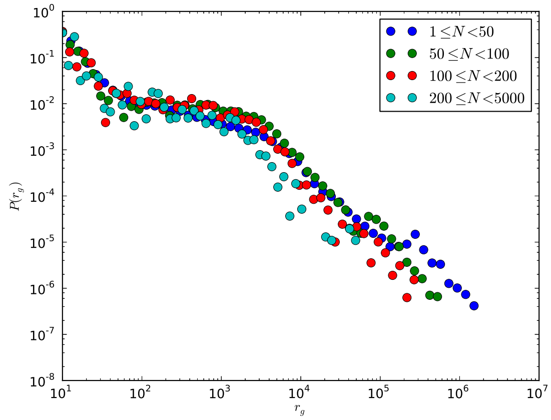

(c)

Figure S1: Impact of the number of tweets per user on observed mobility dynamics: (a) distribution of the number of tweets N per user; (b) displacement distribution for groups of users with different N; (c) distribution of the gyration radius for groups with different N.

number of tweets provides more fine-grained sampling of their actual movement, leading to shorter observed steps between position samples. Figure S1(c) shows the distribution of r_g split across the same user groups. Again, we observe the same three modes of movement regardless of N and the distributions are broadly similar.

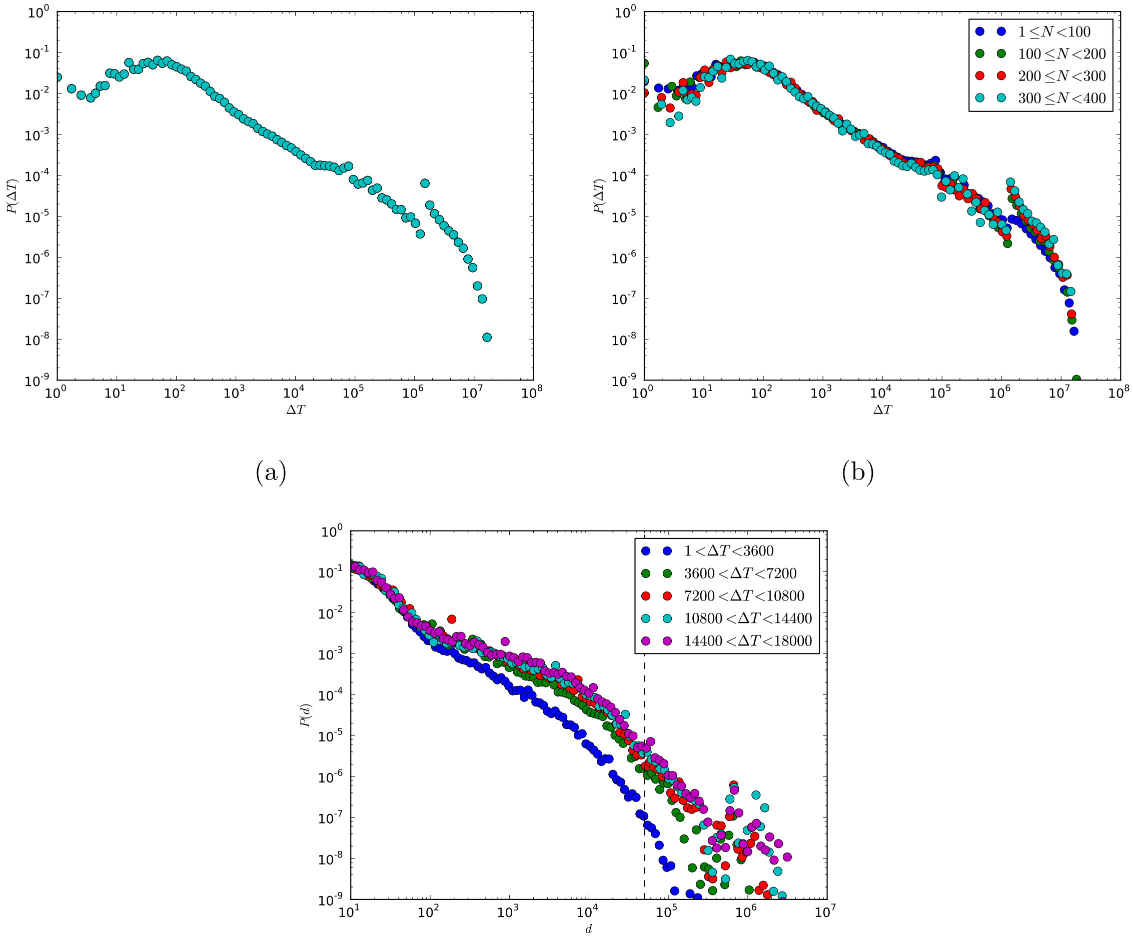

(a) (b)

(c)

Figure S2: Impact of the inter-tweet time on observed mobility dynamics: (a) distribution of the time interval between tweets for the entire population; (b) distribution of the time interval between tweets separated by number of tweets for each user; (c) displacement distribution for groups of users with different ΔT.

We then study the inter-event time of tweeting, i.e. the time interval between a user's two consecutive tweets. As shown in Figure S2(a), the inter-event time distribution also follows a fat-tailed distribution, which indicates, that unlike a homogeneous process with Poissonian

distribution [10, 11], heterogeneous mechanisms or bursty dynamics such as prioritising task execution [8] or reinforcement decision-making [9] may exist in tweeting behaviour. We also observe a discontinuity in the plot around 86,400 corresponding to the day/night cycle. To check whether our results depends on the individual tweeting frequency, we group users into five categories based on their number of tweets and recalculate the inter-event time distribution in each group. Figure S2(b) shows the results where the inter-event time in each group exhibits a similar fat-tailed distribution showing no structural difference compared to the aggregated result for the whole population presented in Figure S2(a). The relatively flat distribution up to about 100 seconds with a minor peak around 1 minute confirms the bursty nature of tweets compared to other modalities such as mobile phones. Finally, we explore the sensitivity of the displacement distribution to inter-event times. We plot the displacement distribution separately for tweets based on the inter event time in Figure S2(c). The distribution for all tweet groups show no structural difference, though the plot for $\Delta T < 3600$ clearly involves shorter displacements. This is expected since users can travel within a bounded distance within one hour of their last tweet, which explains the faster decay of this plot for larger distances.

S2 Technology Dependencies

To explore whether the observed irregularity in the distribution of d is merely due to GPS resolution, knowledge of the error associated with each reported location is important. Zandbergen in [5] reports median errors of 8, 74 and 600 m associated with the locations obtained from an iPhone 3G using respectively Assisted GPS (A-GPS), WiFi positioning and cellular network positioning. However, the integration of High Sensitivity GPS (HSGPS) chipsets in modern mobile phones allows for a relatively consistent availability of a GPS signal. In fact, Zandbergen et al. in [6] reported an availability of valid GPS position fixes on HSGPS-enabled mobile phones close to 100% in different outdoor and indoor settings, and found errors not exceeding 30 m outdoor and 100 m indoor in their measurements. Mobile phones with built-in HSGPS chipsets include devices as old as the iPhone 3GS and Nokia N95 [7], suggesting that the technology is well incorporated in modern cellular phones. This indicates

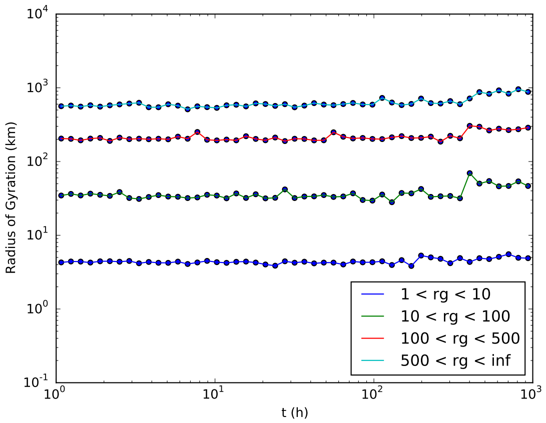

Figure S3: The radius of gyration(km) as a function of time(h)

that the locations reported by mobile phones and that are used in our study are relatively reliable.

S3 Time evolution of r_g

We find that the radius of gyration as a function of time $r_g(t)$, averaged over the whole population, in Figure S3 increases ultra-slowly, which confirms that strong recurrent patterns exist in human mobility. This information is of value for modelling disease risk, for instance, as it indicates that observing the first few hours of tweets can strongly indicate the longer-term r_g for a particular person. Thus, limited empirical data can seed mobility models for initial r_g values of people, and these values remain relatively stable over time.

S4 Visitation frequency for different r_g

We now explore how the visitation frequency changes for users with different r_g, using the same approach as Figure 2. The results are shown in Figure S4 for a cluster size of 250m. Clearly, all r_g groups follows Zipf's law of preferential return, yet the likelihood to be at the most popular location decreases with increasing r_g (see insets). Similarly, the steepness of the plots drops with increasing r_g, indicating that people who move further have lower preference to return to previously visited locations. This effect is likely to result from the

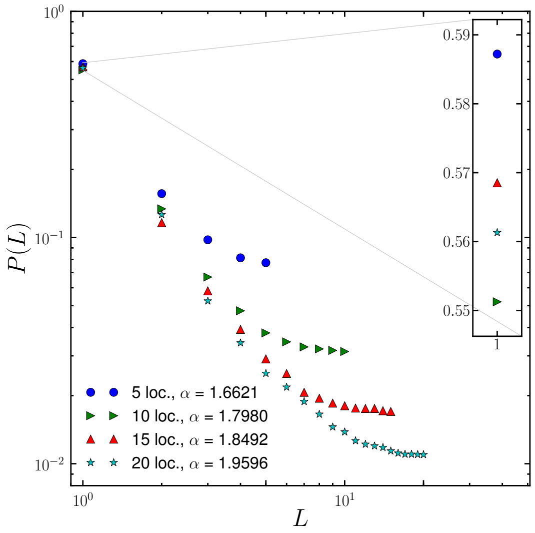

(a) $P(L)$ vs L for users with $1 < r_g < 10$

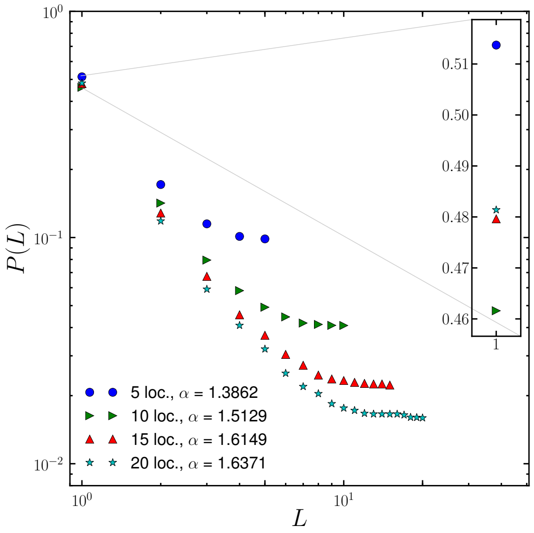

(b) $P(L)$ vs L for users with $10 < r_g < 100$

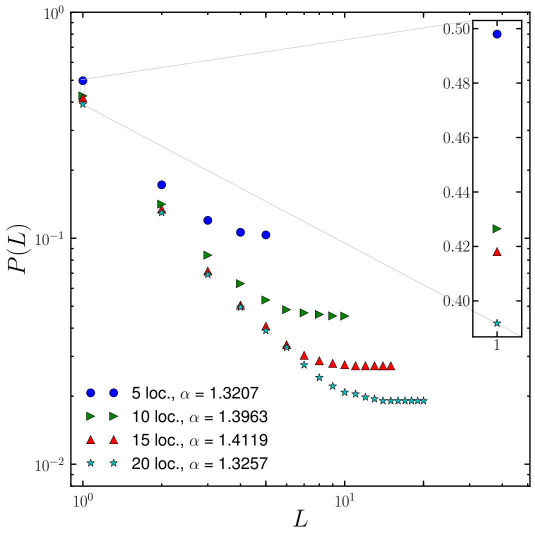

(c) $P(L)$ vs L for users with $100 < r_g < 500$

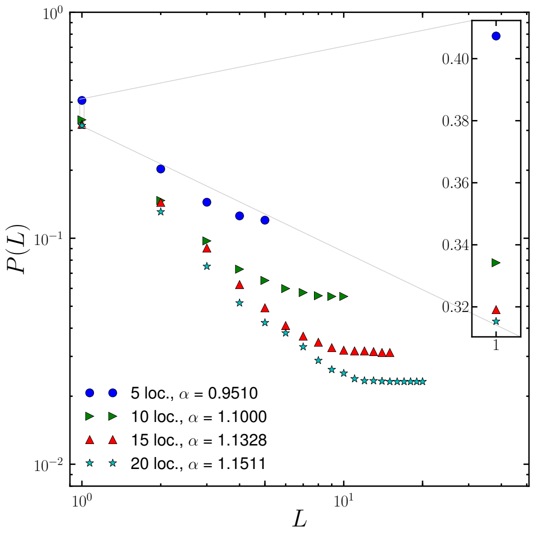

(d) $P(L)$ vs L for users with $r_g > 500$

Figure S4: $P(L)$ vs L for users with different radius of gyration. The inset shows the results for $P(L = 1)$. The α values in the legend show the power law fit exponent.

higher cost [2] people incur for long-distance movement, which firstly increases the return cost, and secondly reduces the perceived value of returning.

S5 Countrywide Tweeting Distributions

Figure S5 shows the density of tweets across all of Australia. Because of the sparse population, the tweet distribution appears extremely sparse in the country that has a comparable area to the continental USA yet with only 23 million people (about 1/15 of the population density). We observe that tweets are mainly clustered around the 3 largest cities in the southeast (Sydney, Melbourne, and Brisbane), with one cluster around Adelaide in the south, another around Perth in the southwest. Lower density areas include the entirety of the east cost of the mainland, and areas around Hobart in the southern island of Tasmania and the city of Darwin in the Northern Territory. The countrywide tweet distributions show similar patterns as in Figure 5, confirming that short and long distance movers remain mainly around the key cities, while intermediate distance movers are more likely to be found further away from key population centres.

S6 Statistical Validation and Goodness of Fit

We use the traditional least squares estimation (LSE) method to get the fitting function of the displacement distribution $P(d)$ and the gyration radius distribution $P(r_g)$. The estimated parameters of the fitting functions for the two fitting schemes in the main text are shown in Table 1-2. Here the probability density function (PDF) of the empirical data is obtained by logarithmic binning [12].

	λ_1	λ_2	β_2	q	R^2	SSE
$P(d)$	0.073 ± 0.002	0.0110 ± 0.0011	0.545 ± 0.010	0.364 ± 0.008	0.999	0.120
$P(r_g)$	0.122 ± 0.005	0.0015 ± 0.0001	0.768 ± 0.011	0.074 ± 0.003	0.997	0.099

Table 1: Fitting with the mixture function indicated by Eq.(1).

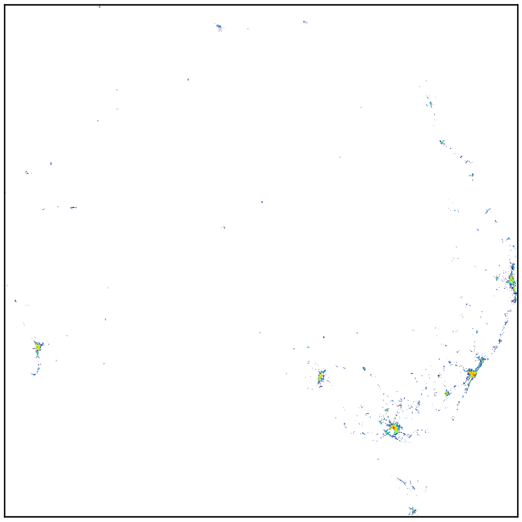

(a) $1 < r_g < 10$

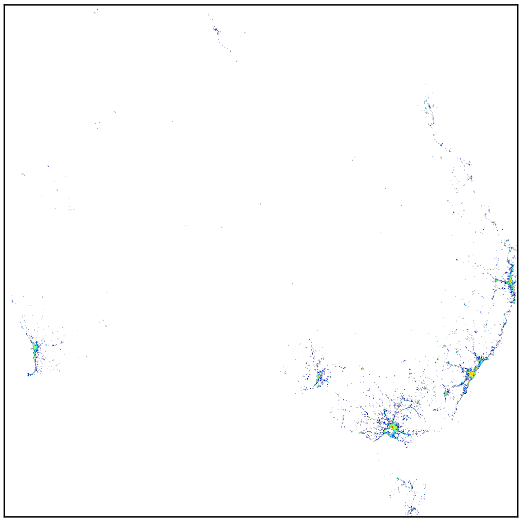

(b) $10 < r_g < 100$

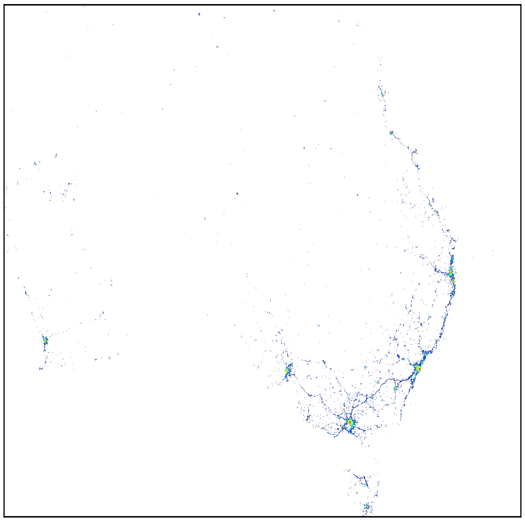

(c) $100 < r_g < 500$

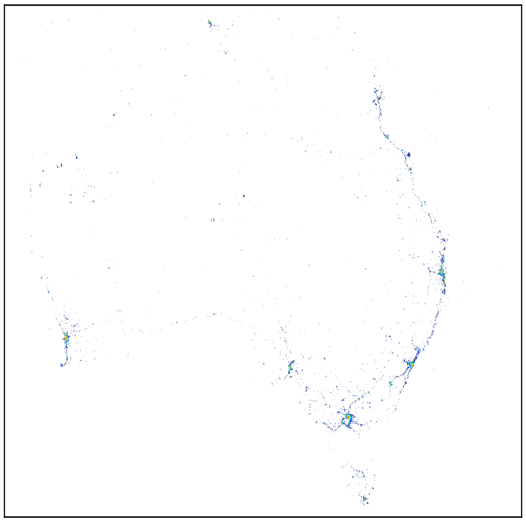

(d) $r_g > 500$

Figure S5: Differences in tweet spatial distributions as the radius of gyration varies for all of Australia. Tweet activity for $1 < r_g < 10$ and $500 < r_g < 1000$ is mainly concentrated in large cities, while tweets for intermediate r_g extend further along main highways and other regions between cities.

	γ_1	γ_2	x_S	R^2	SSE
$P(d)$	0.766 ± 0.012	2.072 ± 0.061	$\approx 10.6km$	0.997	0.086
$P(r_g)$	0.405 ± 0.011	1.600 ± 0.050	$\approx 4.4km$	0.996	0.041

Table 2: Fitting with the double power-law function indicated by Eq.(2).

It is arguable that maximum likelihood estimation (MLE) method is usually more powerful in the estimation of fitting parameters from broad distributions such as a power-law or an exponential [3], especially when the sample size is small. However, using MLE to fit a mixture function of broad distributions is not easy to implement and the performance is not well understood. Indeed, recent studies suggested that, when the sample size is large (e.g. in our study millions of displacements are used for fitting), traditional methods like LSE are comparable to the state-of-the-art methods like MLE [1]. LSE combined with logarithmic binning can even perform better than MLE in some cases [13].

To demonstrate that $P(d)$ with $d \in [100m, 50km]$ corresponding to the regime of urban movements is better approximated by a stretched-exponential compared to other candidate models with a single statistical function such as truncated power-law or log-normal, we use Akaike's information criterion (AIC) [14] to measure the relative goodness of fit for this part. In particular, AIC for each candidate model i is given by

$$AIC_i = -2 \log L_i + 2K_i \tag{1}$$

where L_i is the maximum likelihood of the fitting function whose parameters are estimated by MLE, and K_i is the number of parameters. The Akaike weight, which represents the relative likelihood of each candidate model i, is then given by

$$w_i = \frac{e^{-\Delta_i/2}}{\sum_i e^{-\Delta_i/2}} \tag{2}$$

where $\Delta_i = AIC_i - AIC_{min}$ and $AIC_{min} = \min\{AIC_i\}$. Here we consider five commonly-used statistical functions for heavy-tailed probability density, namely exponential (E), power-law (PL), truncated power-law (TPL), log-normal (LN) and stretched-exponential (SE). It is clear that stretched-exponential has a dominating Akaike weight over other candidate functions, as shown in Table 3.

	E	PL	TPL	LN	SE
w_i	0	0	0	0	1

Table 3: The Alkaike weight for each candidate model.

S7 Clustering Effects

For evaluating return probabilities, the trajectories of the users were adjusted in the following way: Each point (x_i, y_i) of a trajectory was mapped to the point (x_c, y_c) where (x_c, y_c) is the centroid of the cluster containing (x_i, y_i). The results for Figures 2, 4, and S4 use cluster sizes of 250m. Here, we investigate the effect of cluster size on the trends that we observe, in order to establish that these trends are independent of our cluster size selection. We note that most studies that use cellular phone traces for mobility analysis [1, 4] do not define explicit location clusters, as the spatial resolution of this data is based on tower locations, and is typically in the order of 1km. In other words, most mobile-phone based studies have implicit cluster sizes of 1 km. Because Twitter data provides a resolution of up to 10m (the realistic resolution of GPS [15]), Twitter-based mobility analysis requires the explicit clustering positions to account for multiple tweets from the same location. To provide a comparison point with cellular phone data, we consider explicit clustering of 1km, in addition to clusters of 50m and 500m.

Figure S6 plots the variation of the probability of return to the most popular location $P(L = 1)$ and the preferential return exponent α for the 3 cluster size values (50m, 500m, 1000m). Compared with Figure 4(b), the cluster size does not affect the dominant trends in these plots. $P(L = 1)$ consistently decreases and α increases with increasing r_g, pointing to weaker preferential return. $P(L = 1)$ increases by about 0.08 as we increase cluster sizes from 50m to 1km, and α decreases slightly indicating a mild strengthening of preferential return for larger clusters. Despite these scale differences, it is clear that the cluster size selection does not affect the observed trends in weaker preferential return for larger r_g.

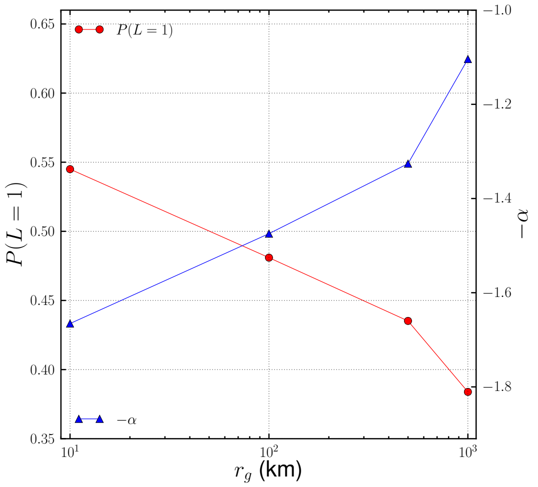

(a) Cluster size = 50m

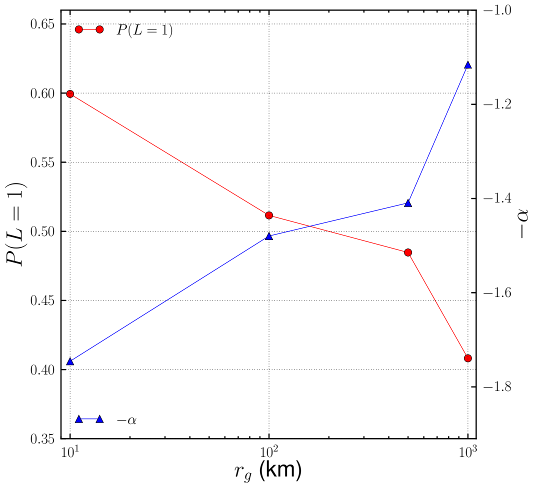

(b) Cluster size = 500m

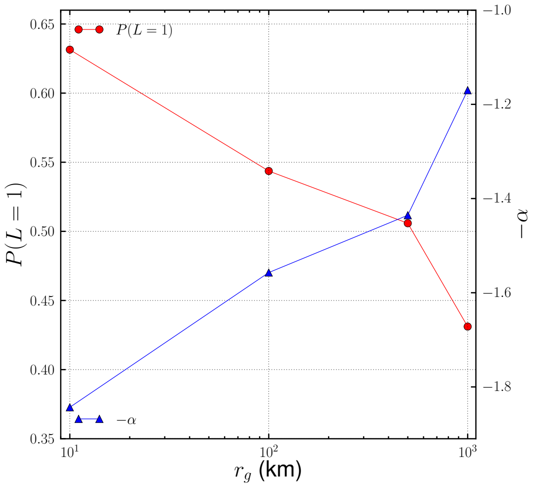

(c) Cluster size = 1000m

Figure S6: The effect of cluster size on observed trends in $P(L = 1)$ and α. Clearly, the cluster size affects the scale but not the pattern of decreasing $P(L = 1)$ and increasing α for larger r_g.

References

[1] Gonzalez, M. C., Hidalgo, C. A., & Barabasi, A. L. Understanding individual human mobility patterns. Nature, **453**(7196), 779-782. (2008)

[2] Yan, X. Y., Han, X. P., Wang, B. H., & Zhou, T. Diversity of individual mobility patterns and emergence of aggregated scaling laws. Scientific reports, **3**. (2013)

[3] Clauset, A., Shalizi, C. R., & Newman, M. E. Power-law distributions in empirical data. SIAM review, **51**(4), 661-703. (2009)

[4] Ji, Y. Understanding human mobility patterns through mobile phone records: a cross-cultural study (Doctoral dissertation, Massachusetts Institute of Technology). (2011)

[5] Zandbergen, P. A. Accuracy of iPhone locations: A comparison of assisted GPS, WiFi and cellular positioning. Transactions in GIS, **13**(s1), 5-25. (2009)

[6] Zandbergen, P. A., & Barbeau, S. J. Positional accuracy of assisted gps data from high-sensitivity gps-enabled mobile phones. Journal of Navigation, **64**(03), 381-399. (2011)

[7] Zhang, J., Li, B., Dempster, A. G., & Rizos, C. Evaluation of high sensitivity GPS receivers. Evaluation. (2010)

[8] Barabasi, A. L. The origin of bursts and heavy tails in human dynamics. Nature, **435**(7039), 207-211. (2005)

[9] Zhao, K., Stehl, J., Bianconi, G., & Barrat, A. Social network dynamics of face-to-face interactions. Physical Review E, **83**(5), 056109. (2011)

[10] Haight, F. A. Handbook of the Poisson distribution. (1967)

[11] Reynolds, P. Call center staffing. The Call Center School Press, Lebanon, Tennessee. (2003)

[12] Newman, M. E. Power laws, Pareto distributions and Zipf's law. Contemporary physics, **46**(5), 323-351. (2005)

[13] Milojevic, S. Power law distributions in information science: Making the case for logarithmic binning. Journal of the American Society for Information Science and Technology, **61**(12), 2417-2425. (2010)

[14] Edwards, A. M. et al. Revisiting Lvy flight search patterns of wandering albatrosses, bumblebees and deer. Nature, **449**(7165), 1044-1048. (2007)

[15] Jurdak, R., Corke, P., Dharman, D., & Salagnac, G. Adaptive GPS duty cycling and radio ranging for energy-efficient localization. In Proceedings of the 8th ACM Conference on Embedded Networked Sensor Systems (pp. 57-70). ACM. (2010, November)
